# HBx Induces the Production of N‐GPC3 in Hepatoma Cells to Promote the Evasion of Macrophage Phagocytosis Through Activating Furin

**DOI:** 10.1111/jcmm.71122

**Published:** 2026-04-09

**Authors:** Jiaying Wu, Hongjiu Yu, Yuli Zhou, Yaoquan Lin, Kaiqi Zhang, Wei Li, Zhongzheng Liu, Mengsen Li, Lianghui Gao

**Affiliations:** ^1^ Key Laboratory of Emergency and Trauma of Ministry of Education, Department of Hepatopancreatobiliary Surgery The First Affiliated Hospital, Hainan Medical University Haikou Hainan Province China; ^2^ Key Laboratory of Tropical Translational Medicine, Ministry of Education, and Hainan Provincial Key Laboratory of Carcinogenesis and Intervention Hainan Medical University Haikou Hainan Province China

**Keywords:** GPC3, HBx, HCC, immune escape, macrophage

## Abstract

The hepatitis B virus X protein (HBx) is a central oncogenic driver in hepatocellular carcinoma (HCC). Although glypican‐3 (GPC3) is a phosphatidylinositol proteoglycan‐specific biomarker for HCC. However, the combined role of HBx and GPC3 in mediating HCC cell evasion from macrophage phagocytosis remains unclear. Here, we demonstrate that HBx modulates macrophage phagocytosis by regulating GPC3 membrane morphology. Analysis of tissues from 30 patients with hepatocellular carcinoma revealed concomitant upregulation of HBx and GPC3 in tumour specimens. Mechanistically, HBx alters ERK site phosphorylation, thereby influencing the enzymatic activity of Furin protease—which cleaves GPC3—and consequently orchestrating morphological changes of membrane‐associated GPC3. To functionally validate this, THP‐1 cells were polarized to M1 macrophages and co‐cultured with HCC cells. Notably, HBx directly impaired macrophage phagocytosis of HCC cells, an effect mediated through GPC3 morphological dynamics. Collectively, these findings indicate that HBx suppresses macrophage phagocytosis of HCC cells by remodelling membrane‐associated GPC3.

**Abbreviations**
GPC3: Glypican‐3
HBV: Hepatitis B virus
HBx: Hepatitis B virus X proteins
HCC: Hepatocellular carcinoma
N‐GPC3: N‐terminal fragment Glypican‐3
TAMs: Tumour‐associated macrophages
TME: The tumour microenvironment

## 1 Introduction

Hepatocellular carcinoma (HCC) is the second leading cause of cancer‐related mortality worldwide exhibiting a five‐year survival rate of approximately 11% [1] Hepatitis B virus (HBV) infection is a major etiological factor for HCC, with its key oncogenic mechanisms encompassing the direct activation of cancer‐related signalling pathways, immune evasion and remodelling of the liver immune microenvironment. Among these, the Hepatocellular B virus X protein (HBx) encoded by HBV functions as the core oncogenic driver in hepatocarcinogenesis [2] HBx constitutively activates key oncogenic signalling pathways, including PI3K/AKT and MAPK/ERK, endowing hepatocytes with malignant transformation traits including uncontrolled proliferation, apoptosis resistance and invasive properties, ultimately leading to hepatocarcinogenesis [3, 4, 5] Clinical cohort studies further demonstrate that individuals with persistent HBx expression face a cumulative 20‐year HCC risk of 40% [6, 7] Its application in HCC has demonstrated limited efficacy, with most patients prone to developing resistance [8, 9] Therefore, defining the molecular mechanisms underlying HBx‐driven HCC progression is critical for improving targeted therapies.

Glypican‐3 (GPC3) is a membrane‐anchored heparan sulphate proteoglycan whose 70‐kDa core protein is cleaved by Furin to generate a 40‐kDa N‐terminal fragment (N‐GPC3) and a 30‐kDa C‐terminal fragment (C‐GPC3), thereby forming three distinct membrane‐associated subtypes, among which N‐GPC3 serves as a serum diagnostic marker for early‐stage hepatocellular carcinoma [10, 11, 12, 13, 14] In normal tissues, GPC3 is scarcely expressed, whereas it is markedly overexpressed in approximately 70% of HCC cases. Furthermore, its positive expression is closely associated with poor patient prognosis. GPC3 is scarcely expressed in normal tissues but markedly upregulated in approximately 70% of HCC cases, where its expression correlates with poor prognosis, thereby establishing GPC3 as a key diagnostic biomarker and a promising immunotherapeutic target in HCC [15, 16, 17] Accordingly, GPC3 has emerged as a key diagnostic biomarker and a promising target for immunotherapy in HCC. Functionally, GPC3 can bind chemokines and cytokines through the positively charged domain of its heparan sulphate chains, remodelling the immunosuppressive microenvironment of tumours. It can also directly modulate macrophage recruitment, thereby playing a pivotal role in the development and progression of cancers such as HCC [18, 19, 20, 21, 22].

Tumour‐associated macrophages (TAMs) are the most abundant immune cells within the tumour microenvironment (TME), comprising over 50% of the cellular infiltrate and playing essential roles throughout tumour progression [23, 24, 25] The functional status of TAMs directly impacts the efficiency of tumour immune clearance: enhancing the phagocytic activity of macrophages against tumour cells has been validated as a highly promising therapeutic strategy [26, 27]. Studies in animal models have shown that targeted inhibition of TAM infiltration can directly delay tumour progression [28] Given the role of GPC3 in modulating macrophage behaviour and the central importance of macrophages in tumour immune evasion, a functional interplay between these factors is likely. However, the association between HBx and GPC3 and their coordinated regulation of macrophage function in HCC remains poorly defined.

In this study, we investigated the regulatory mechanisms involving HBx, GPC3 and macrophages during tumour development and progression.

## 2 Materials and Methods

### 2.1 Cell Lines and Culture

The human hepatocellular carcinoma cell lines Huh7 and PLC were cultured in DMEM supplemented with 10% FBS and 1% penicillin–streptomycin, whereas the human monocytic cell line THP‐1 was maintained in RPMI 1640 containing 10% FBS and 1% penicillin–streptomycin. Cell line authentication was performed by short tandem repeat (STR) profiling, confirming consistency with reference profiles in the Chinese Cell Line Resource Infrastructure. Mycoplasma contamination was routinely assessed by PCR, and all cell lines tested negative.

### 2.2 Tissue Microarray and Immunohistochemistry

A total of 30 paired HCC tumour tissues and corresponding adjacent non‐tumour tissues were used to construct tissue microarrays. All tissue samples were obtained from the First Affiliated Hospital of Hainan Medical University, with all enrolled patients being those undergoing liver resection for the first time. All HCC samples used in this study have obtained authorization from the Research Ethics Review Committee of Hainan Medical University, and the study protocol has also been approved by this ethics committee (Ethics Review No.: HYLL‐2024‐827). Immunohistochemistry detection was performed by Servicebio in accordance with standard protocols, and the antibodies used in the experiment included HBx antibody (R&D systems, USA) and GPC3 antibody (Abcam, USA).

### 2.3 Multiplexed Immunohistochemistry

Multiplex immunohistochemical staining was performed using a commercial kit (Absin, abs50030) according to the manufacturer's instructions: tissue microarrays were deparaffinized, rehydrated and subjected to microwave antigen retrieval, followed by blocking with 10% normal goat serum, incubation with primary antibodies against HBx and GPC3, TSA‐based signal amplification, DAPI nuclear counterstaining and image analysis using KFslideOS software. 2.4 Lentiviral infection and screening of stable expression cell lines.

Lentiviral transduction was used to generate HBx‐overexpressing Huh7 cells, HBx‐silenced PLC cells and GPC3‐silenced HBx‐overexpressing Huh7 cells. The lentiviral vector for HBx overexpression carried the target HBx gene and a fluorescent marker gene, the lentiviral vector for HBx knockdown carried specific shRNA sequences targeting HBx, and the lentiviral vector for GPC3 knockdown carried specific shRNA sequences targeting GPC3. All three lentiviruses were designed and packaged by Yunzhou Bio. At 48h–72 h post‐transfection, transfection efficiency was observed via fluorescence microscopy (ensuring a positive rate ≥ 80%), followed by monoclonal selection in 6‐well plates using selection medium containing 0.5 g/mL puromycin. Western blot was used to detect the expression levels of HBx protein and GPC3.

### 2.4 Western Blot

Cells were lysed on ice with pre‐cooled RIPA lysis buffer (containing protease inhibitors, phosphatase inhibitors and PMSF), after which total proteins were extracted and their concentrations were determined. The extracted proteins were separated by SDS‐PAGE gel electrophoresis and then electrotransferred onto PVDF membranes (Merck Millipore, USA). The membranes were blocked with serum‐free rapid blocking buffer at room temperature, followed by incubation with corresponding primary antibodies overnight at 4°C. The primary antibodies used included HBx (R&D Systems, USA), GPC3 (Abcam, USA), Furin (Huabio, China), ERK (Huabio, China), p‐ERK (Huabio, China) and GAPDH (Proteintech Group, USA). On the following day, secondary antibodies (Proteintech Group, USA) were incubated at room temperature, and protein signals were detected using an ECL chemiluminescence kit.

### 2.5 Inducing Macrophage Polarization

THP‐1 cells were first centrifuged at 800 rpm for 5 min. After centrifugation, the supernatant was discarded, and then fresh medium containing 50 ng/mL phorbol ester was added (the volume was sufficient to resuspend the cells thoroughly) to adjust the cell concentration to 1 × 10^6^/mL. The cells were gently pipetted to form a suspension, then seeded into 6‐well plates at a volume of 2 mL per well, and cultured in a 37°C, 5% CO_2_ incubator in the dark. After 48 h of PMA treatment, THP‐1 cells exhibited adherent morphology with pseudopod formation, indicating differentiation into M0 macrophages. Subsequently, M0‐type macrophages were washed twice with an appropriate amount of sterile PBS (PBS was discarded after each wash), and the medium was replaced with fresh medium containing 50 ng/mL LPS + 20 ng/mL IFN‐γ. Cultivation was continued for 24 h, and M1‐type macrophages were finally obtained.

### 2.6 Flow Cytometry

M1‐polarized macrophages were serum‐starved for 2 h prior to co‐culture; after treatment with Accutase digestive enzyme for 10 min, the cell suspension was collected and counted using a haemocytometer. Meanwhile, lentivirus‐transfected hepatocellular carcinoma cells were taken and mixed with M1‐type macrophages at a ratio of 1:1. Fresh serum‐free medium was added to adjust the total volume of the cell suspension to 2 mL per EP tube, and the co‐culture was performed in a 37°C, 5% CO_2_ incubator in the dark for 2 h. After completion, a pipette was used to thoroughly pipette the bottom of the well to collect all cells. The cell suspension was filtered through a 300‐mesh cell sieve, and the filtered cell suspension was then transferred to a flow cytometry tube for on‐machine detection.

### 2.7 Immunofluorescence

M1‐type macrophages were polarized directly in confocal dishes and then subjected to starvation treatment. Hepatocellular carcinoma cells were labelled with 0.5 μL of 5 mM CFDA‐SE at 37°C for 20 min in the dark, washed twice with PBS, and subsequently counted. The above‐labelled hepatocellular carcinoma cells were mixed with M1‐type macrophages at a ratio of 1:1 and co‐cultured in the dark for 2 h. After co‐cultivation, the cells were washed twice with PBS to remove unphagocytosed cells and fixed with 4% paraformaldehyde. Immunoblocking was performed after fixation. All subsequent operations were carried out in the dark: primary antibodies, fluorescent secondary antibodies, and DAPI staining solution were added in sequence. Finally, anti‐bleaching agent was added for mounting, and the samples were wrapped in tin foil and stored at 4°C for short‐term or−20°C for long‐term. Images of the samples were collected using a laser confocal microscope.

### 2.8 Cell Migration Assay

Macrophages were dissociated with Accutase and resuspended in serum‐free medium at a density of 1 × 10^6^ cells/mL. Transwell chambers (pore size, 8 μm) were used, with 500 μL of fresh RPMI 1640 medium containing 20% FBS added to each well of the lower chamber. 500 μL of cell suspension was added to each upper chamber, and the cells were cultured in a cell incubator for 48 h. Cells in the upper layer of the Transwell chamber were gently wiped off with cotton balls, washed twice with PBS, and treated with 4% paraformaldehyde for 20 min. The fixed Transwell chambers were cleaned twice with PBS and stained with 0.1% crystal violet for 20 min. After staining, residual staining solution was washed off with PBS, and the plates were allowed to dry. Images were captured under an inverted microscope, and five random fields of view were selected for each group to calculate the average value.

### 2.9 Incucyte Live Cell Imaging Analyser System

THP‐1 cells were seeded in 96‐well plates at a density of 3–5 × 10^5^/mL, polarized into M1‐type macrophages, and then subjected to starvation treatment for 2 h. Hepatocellular carcinoma cells were resuspended in labelling buffer to 1 × 10^6^/mL, aliquoted, labelled at 37°C for 1 h, washed with complete medium, and maintained at the original density. Subsequently, macrophages and labelled hepatocellular carcinoma cells were co‐cultured at a 1:1 ratio, with three replicate wells per group. Three random fields of view were selected per well, and images were captured every hour to continuously record changes in fluorescent signals for 24 h.

### 2.10 Live Cell Imager

THP‐1 cells were seeded in 6‐well plates at a density of 3–5 × 10^5^ cells/mL, induced to differentiate into M1 macrophages, and serum‐starved for 2 h. After digestion, hepatocellular carcinoma cells were washed twice with PBS. Huh7 and PLC cells were centrifuged at 1300 rpm and 1600 rpm, respectively, for 5 min. Hepatocellular carcinoma cells and macrophages were co‐cultured in complete medium at a ratio of 1:1, with images captured every hour to continuously record dynamic changes over 24 h. Five different fields of view were randomly selected for each group, with images captured every 30 min for 24 h.

### 2.11 Statistical Analysis

Each experiment was repeated more than three times. Statistical analysis was performed using GraphPad Prism 9. All data were expressed as mean ± standard deviation. The t–test was applied to determine the statistical significance of differences between two independent groups. One‐way analysis of variance was used to compare differences among multiple groups, and the data were expressed as mean ± standard deviation (x ± SD). A difference was considered statistically significant when *p* < 0.05.

## 3 Results

### 3.1 High Expression of HBx, GPC3 and CD86 in HCC Tissues

**FIGURE 1:**
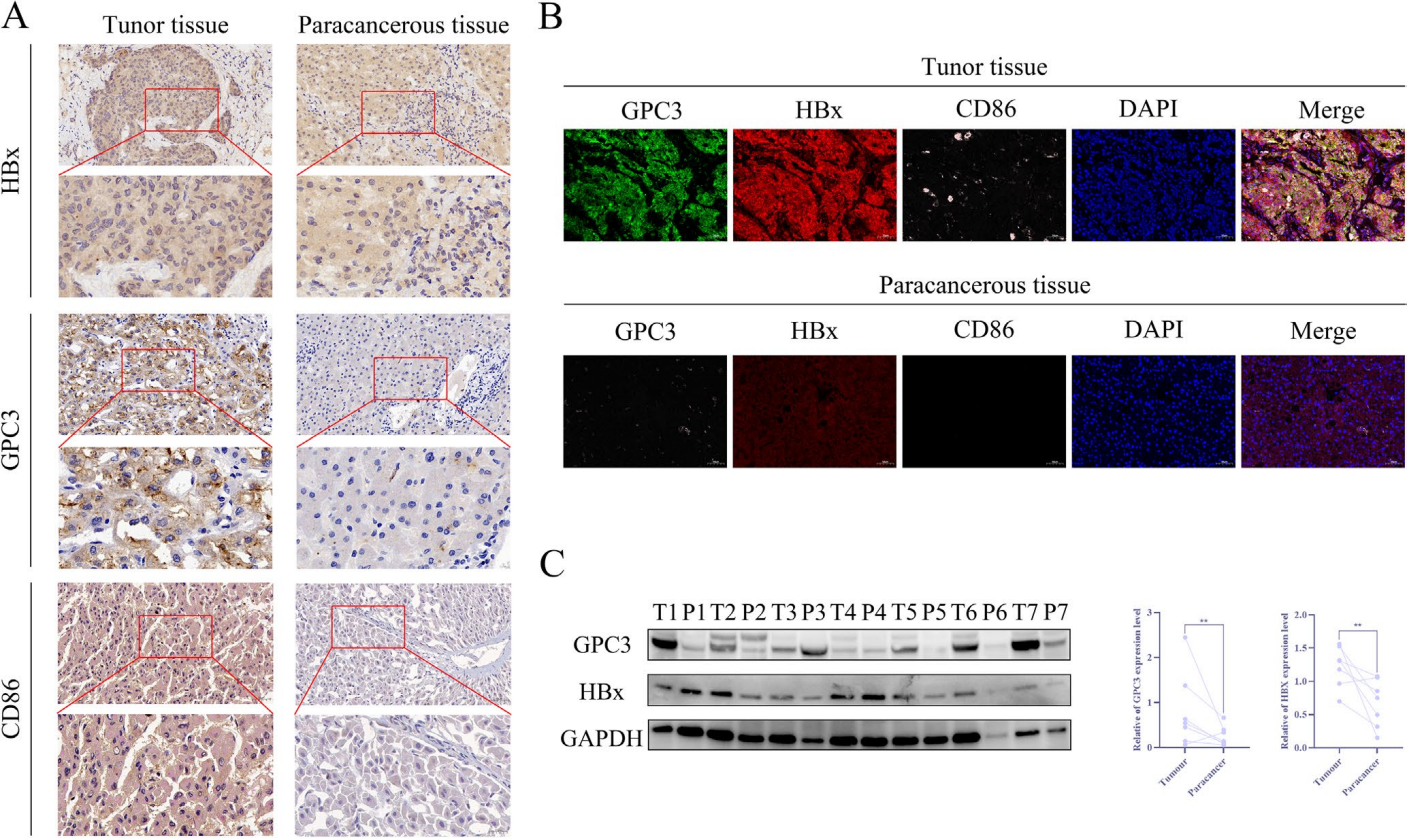
Clinical significance of HBx, GPC3 and CD86 expression in HCC (A) Identification of differential expression of HBx, GPC3 and CD86 in 30 cases of HCC tissues and adjacent tissues by immunohistochemical staining; (B) Detection of localization of HBx, GPC3 and CD86 by immunofluorescence assay; (C) Detection of differential expression levels of HBx and GPC3 in HCC and adjacent tissues by Western blotting.

To assess the clinical relevance of HBx, GPC3 and CD86 in HCC, immunohistochemical analyses were performed on tissue microarrays comprising 30 paired HCC and adjacent non‐tumour samples. The results showed that positive staining densities for HBx, GPC3 and CD86 in tumour tissues were significantly higher than in normal liver tissues. Moreover, compared to the HBx‐negative group, the expression levels of GPC3 and CD86 were markedly increased in HBx‐positive tumours (Figure 1A). Immunofluorescence revealed that HBx was primarily localized in the cytoplasm of hepatoma cells, whereas GPC3 was specifically highly expressed on the cell membrane (Figure 1B). Western blotting analysis of seven paired clinical samples further confirmed that the protein expression levels of HBx and GPC3 in HCC tissues were significantly higher than in the corresponding adjacent non‐tumour tissues (Figure 1C). In summary, HBx, GPC3 and CD86 exhibit synergistic high expression and distinct subcellular localization in hepatocellular carcinoma, suggesting a potential functional interaction among the three in hepatocarcinogenesis and progression. These findings provide a clinical rationale and research direction for further exploring whether HBx influences the tumour immune microenvironment by regulating GPC3.

### 3.2 HBx Promotes the Proliferation and Migration of Hepatoma Cells

**FIGURE 2:**
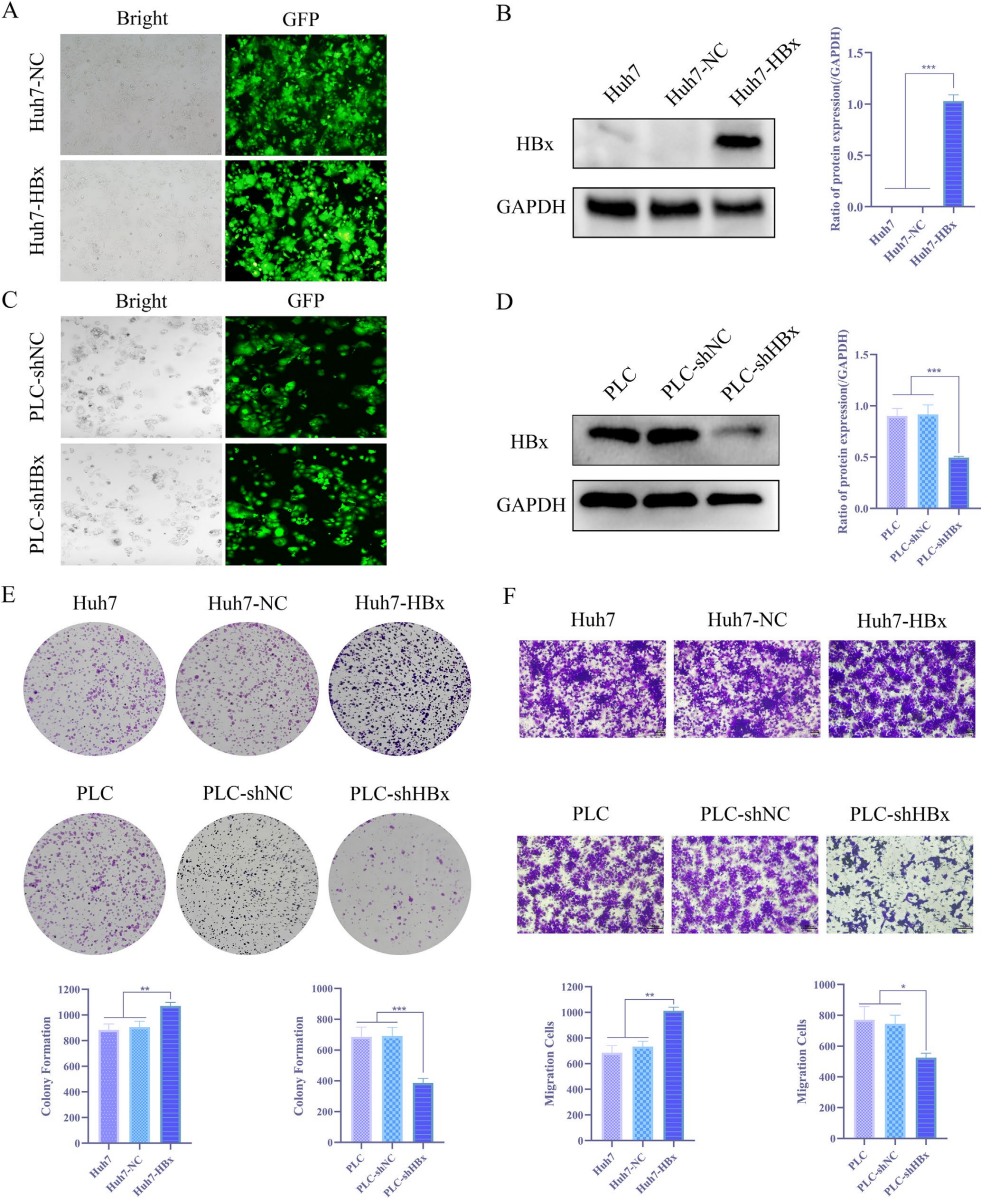
HBx significantly promotes the proliferation and migration of hepatocellular carcinoma cells (A) Observation of lentivirus transfection in Huh7 cells by fluorescence microscopy; (B) WB assay to detect the content of HBx in Huh7, Huh7‐NC and Huh7‐HBx; (C) Observation of lentivirus transfection in PLC cells by fluorescence microscopy; (D) WB assay to detect the content of HBx in PLC, PLC‐shNC and PLC‐shHBx; (E) Effect of HBx on the cloning ability of Huh7 cells and PLC cells; (F) Effect of HBx on the migration ability of Huh7 cells and PLC cells.

To elucidate the regulatory role of HBx in the malignant phenotype of hepatocellular carcinoma cells, this study employed lentiviral transfection technology to establish stable HBx‐overexpressing and HBx‐knockdown cell models in HCC cell lines, which were used as reciprocal controls in the experiments (Figure 2A–D). Using these models, we examined the effect of HBx on cell proliferation and migration. Collectively, these data demonstrate that HBx directly promotes the proliferative and migratory potential of HCC cells, supporting its critical pro‐oncogenic role in HCC progression and providing a functional basis for subsequent mechanistic studies (Figure 2E,F). These findings collectively demonstrate that HBx can directly regulate the proliferative and migratory activities of HCC cells, suggesting its crucial pro‐oncogenic function in HCC progression and providing a functional foundation for subsequent in‐depth investigation of its molecular mechanisms.

### 3.3 Overexpression of HBx Upregulates the Expression of N‐GPC3 in Hepatoma Cells

**FIGURE 3:**
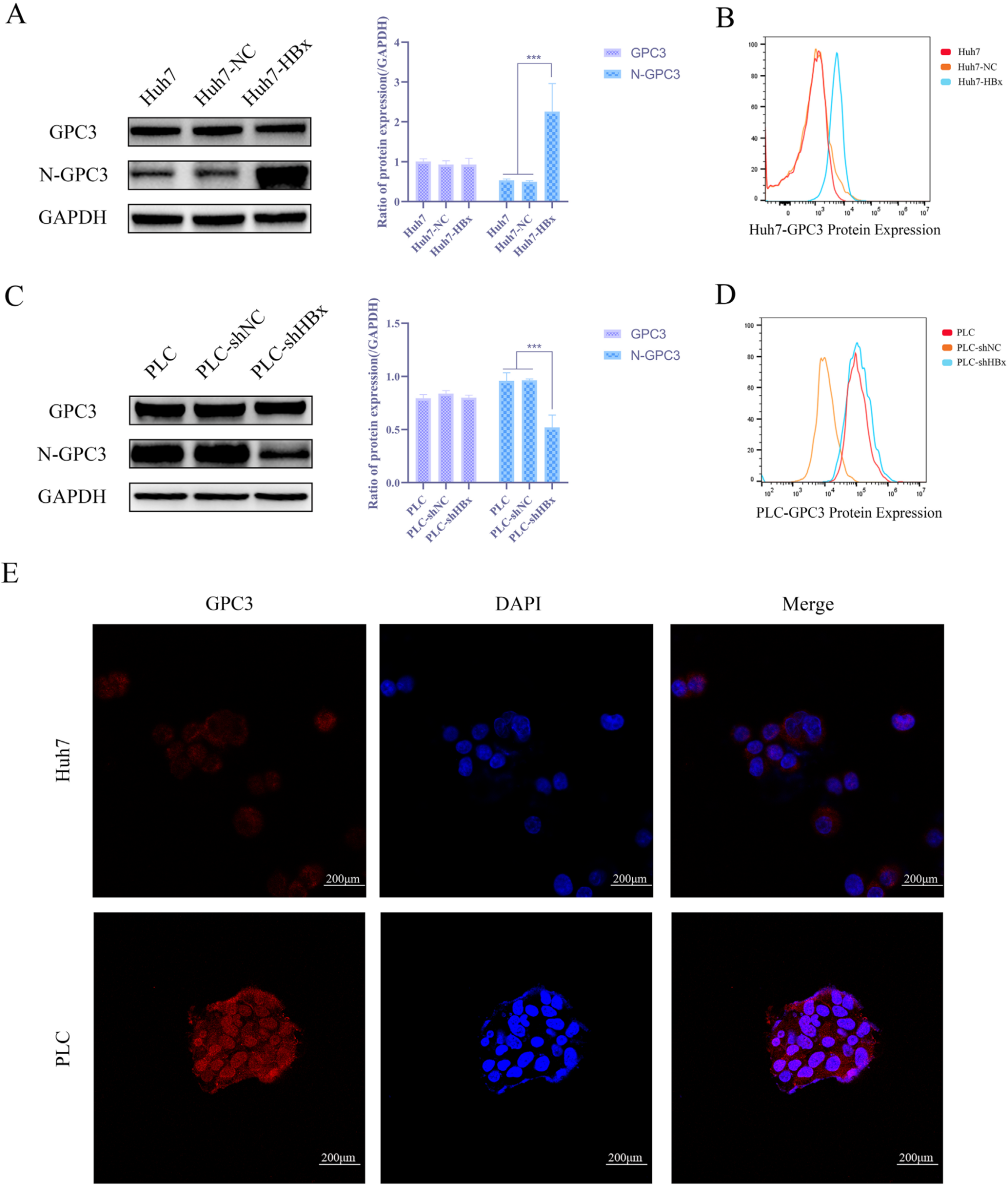
Overexpression of HBx upregulates N‐GPC3 expression in hepatocellular carcinoma cells (A) WB assay to detect the expression levels of GPC3 and N‐GPC3 in Huh7, Huh7‐NC and Huh7‐HBx; (B) Flow cytometry to detect the total GPC3 expression on the cell membrane of Huh7, Huh7‐NC and Huh7‐HBx with overexpression; (C) WB assay to detect the contents of GPC3 and N‐GPC3 in PLC, PLC‐shNC and PLC‐shHBx; (D) Flow cytometry to detect the total GPC3 expression on the PLC cell membrane of PLC, PLC‐shNC and PLC‐shHBx; (E) Immunofluorescence assay to detect the localization of GPC3 in Huh7 cells and PLC cells.

To further clarify the regulatory effect of HBx on GPC3 expression, this study, building on the previously observed correlation between their expression in HCC tissues, focused on analysing the impact of HBx on different forms of GPC3. Western blot results showed that HBx overexpression in Huh7 cells significantly upregulated the expression of the 40 kDa N‐GPC3, while HBx knockdown in PLC cells inhibited N‐GPC3 levels. Notably, total full‐length GPC3 protein abundance remained unchanged despite alterations in HBx expression (Figure 3A,C). Flow cytometry also indicated that HBx overexpression significantly increased the overall expression of GPC3 on the surface of Huh7 cells, whereas HBx knockdown reduced it on PLC cells (Figure 3B,D). Immunofluorescence assays confirmed that GPC3 is primarily localized on the cell membrane (Figure 3E). Notably, total full‐length GPC3 protein abundance remained unchanged despite alterations in HBx expression. This provides important clues for elucidating the potential mechanism by which HBx regulates the tumour microenvironment through GPC3.

### 3.4 HBx Increases Furin Enzyme Content and Promotes Membrane N‐GPC3 Expression

**FIGURE 4:**
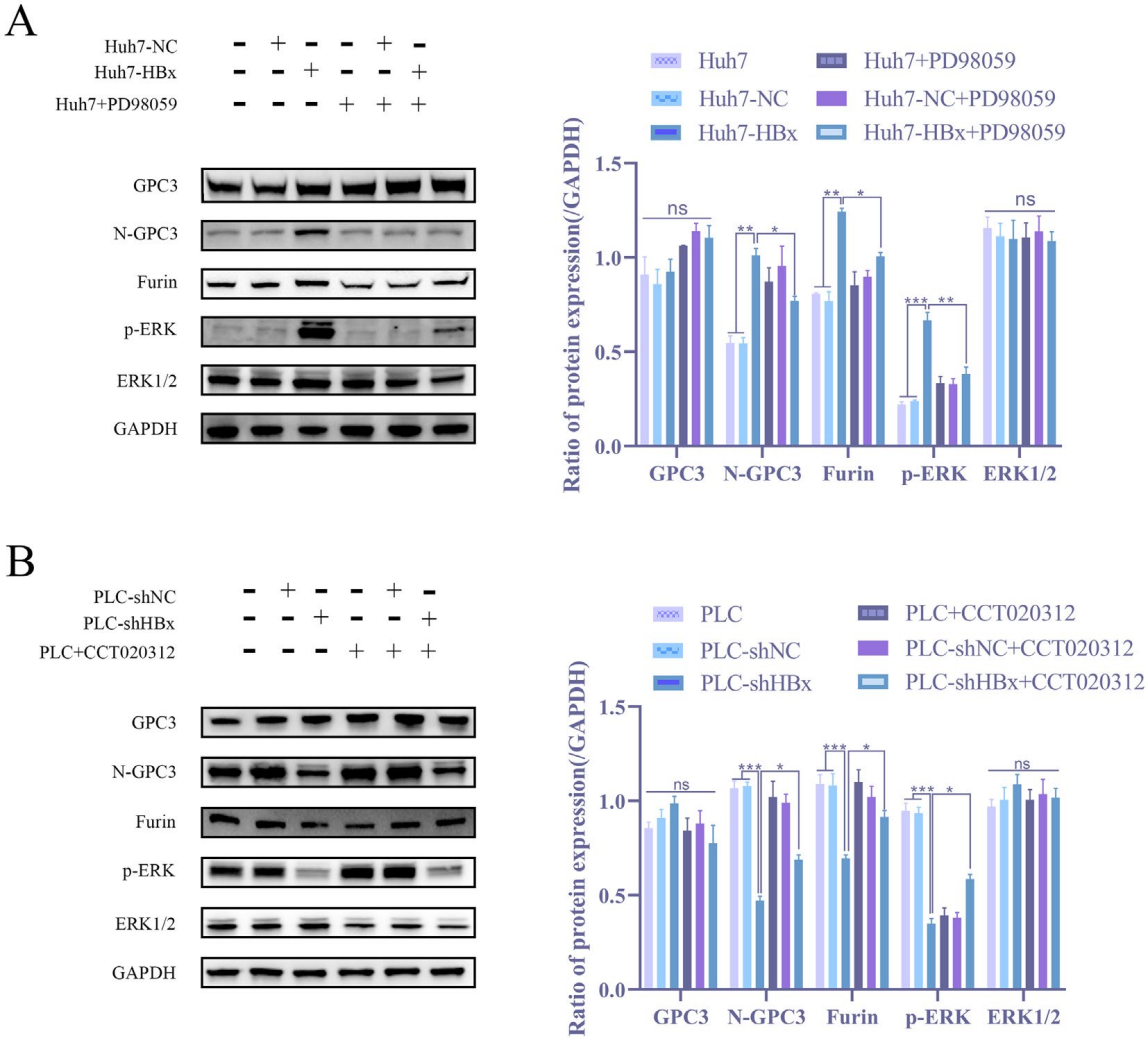
HBx increases Furinase content and promotes membrane N‐GPC3 expression (A) WB assay to detect protein changes of GPC3, N‐GPC3, Furin, p‐ERK and ERK in Huh7, Huh7‐NC, Huh7‐HBx, as well as in Huh7, Huh7‐NC and Huh7‐HBx before and after the addition of MAPK pathway inhibitor PD98059; (B) WB assay to detect protein expression changes of GPC3, N‐GPC3, Furin, p‐ERK and ERK in PLC, PLC‐shNC, PLC‐shHBx, as well as in PLC, PLC‐shNC and PLC‐shHBx before and after the addition of MAPK pathway agonist CCT020312.

Building on our finding that HBx selectively promotes N‐GPC3 expression and previous reports linking p‐ERK to HBx and Furin [29, 30] we investigated whether HBx regulates GPC3 processing through the MAPK/ERK pathway. Pharmacological modulation of MAPK signalling was performed using the inhibitor PD98059 and the agonist CCT020312. The results showed that in Huh7 cells, HBx overexpression significantly upregulated p‐ERK levels and concurrently increased the expression of both Furin and N‐GPC3, while PD98059 treatment inhibited these effects (Figure 4A). Conversely, in PLC cells, HBx knockdown reduced the expression of p‐ERK, Furin and N‐GPC3, whereas CCT020312 partially restored p‐ERK activity (Figure 4B). Total GPC3 protein levels did not change significantly under any condition. These findings indicate that HBx promotes Furin expression by activating the MAPK/ERK pathway, thereby enhancing the cleavage of GPC3, ultimately leading to a specific increase in membrane‐bound N‐GPC3. This mechanism reveals a key role for the HBx‐ERK‐Furin axis in regulating the functional form of GPC3, providing new molecular insights into how HBx influences the tumour microenvironment.

### 3.5 HBx Significantly Promotes Hepatoma Cells to Escape Phagocytosis by Macrophages

**FIGURE 5:**
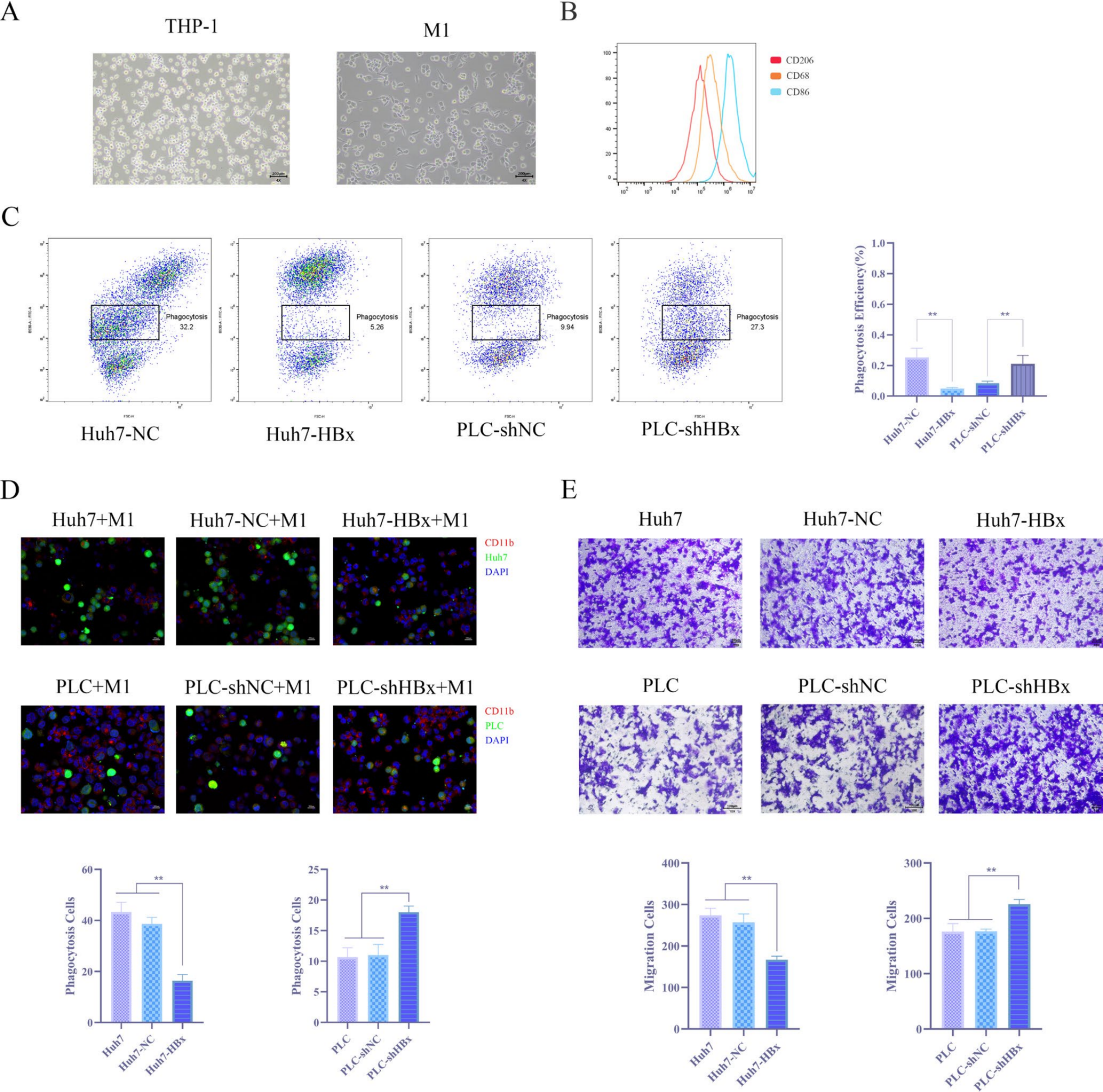
HBx significantly promotes phagocytosis of hepatocellular carcinoma cells escaping macrophages (A) Microscopic observation of the morphology of THP‐1 and M1‐type macrophages; (B) Flow cytometry to detect the expression levels of macrophage markers CD86, CD68 and CD206; (C) Flow cytometry to detect the proportion of Huh7‐NC, Huh7‐HBx, PLC‐shNC and PLC‐shHBx phagocytosed by macrophages; (D) Immunofluorescence assay to detect the effect of HBx on the ability of hepatoma cells to escape macrophages; (E) Transwell chamber assay to detect the effect of HBx on the ability of hepatoma cells to attract macrophage migration.

**FIGURE 6:**
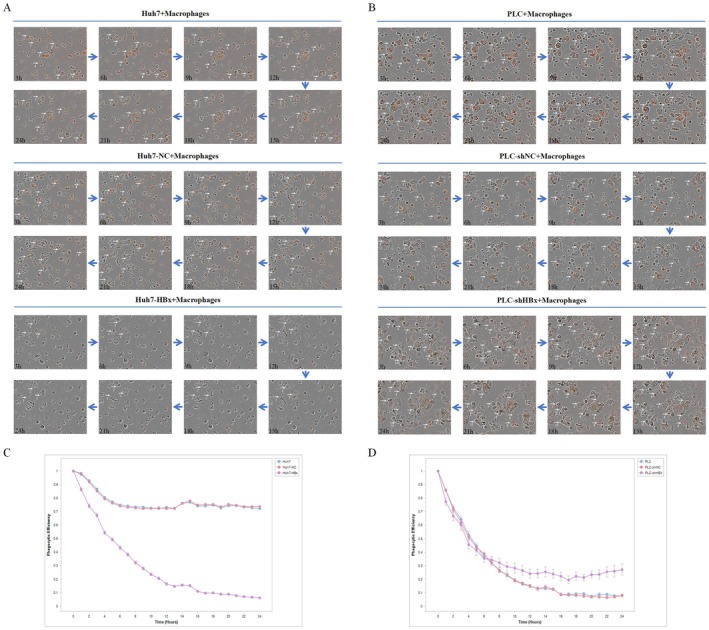
Detection of macrophage phagocytosis by Incucyte live cell imaging analysis system (A) Incucyte live cell imaging analysis system to track the phagocytic degree of macrophages on Huh7, Huh7‐NC and Huh7‐HBx cells; (B) Incucyte live cell imaging analysis system to track the phagocytic degree of macrophages on PLC, PLC‐shNC and PLC‐shHBx cells; (C) Incucyte live cell imaging analysis system to analyse the phagocytic rate of macrophages on Huh7 cells; (D) Incucyte live cell imaging analysis system to analyse the phagocytic rate of macrophages on PLC cells.

To determine the direct effect of HBx on the immune evasion capability of hepatocellular carcinoma cells, this study established a co‐culture system of HCC cells and M1‐type macrophages (Figure 5A). First, THP‐1 cells were induced with PMA to polarize into the M1 phenotype, which was validated by high expression of CD86 (Figure 5B). Quantitative detection by flow cytometry revealed that, compared to the control group, the phagocytic rate of HBx‐overexpressing Huh7 cells by macrophages was significantly reduced, whereas the phagocytic rate of HBx‐knockdown PLC cells was significantly increased (Figure 5C). Immunofluorescence assays visually confirmed this difference morphologically (Figure 5D). Further investigation using Transwell assays to assess directed cell migration showed that HBx overexpression inhibited the migration of macrophages toward HCC cells, while HBx knockdown promoted this migration (Figure 5E). The Incucyte live‐cell imaging analysis system was used to track the phagocytosis of individual HCC cells by macrophages and quantify changes in fluorescence intensity. The results similarly demonstrated that HBx overexpression suppressed macrophage phagocytic activity, whereas HBx knockdown enhanced it (Figure 6A–D). In summary, multiple lines of evidence consistently indicate that HBx inhibits the phagocytosis of and migration toward HCC cells by macrophages, thereby functionally confirming its role in promoting tumour immune evasion.

### 3.6 Knockdown of GPC3 Expression Enhances Macrophage Phagocytosis Inhibited by HBx

**FIGURE 7:**
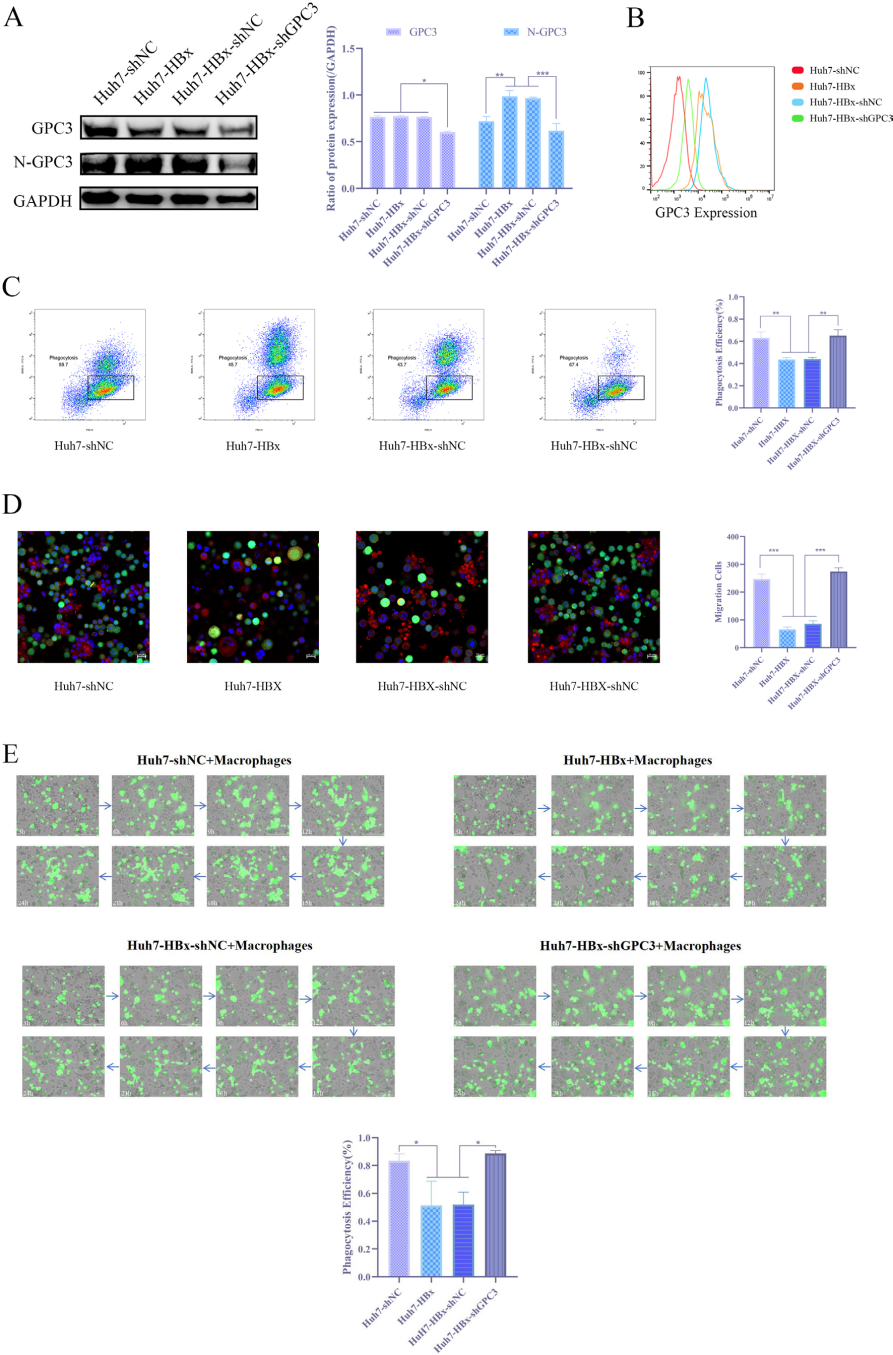
Knockdown of GPC3 expression enhances phagocytosis in macrophages inhibited by HBx (A) WB assay to detect the contents of GPC3 and N‐GPC3 in Huh7‐shNC, Huh7‐HBx, Huh7‐HBx‐shNC and Huh7‐HBx‐shGPC3; (B) Flow cytometry to detect the expression of total GPC3 on the cell membrane of Huh7‐shNC, Huh7‐HBx, Huh7‐HBx‐shNC and Huh7‐HBx‐shGPC3; (C) Flow cytometry to detect the phagocytic rate of macrophages on Huh7‐shNC, Huh7‐HBx, Huh7‐HBx‐shNC and Huh7‐HBx‐shGPC3; (D) Immunofluorescence to detect the capture rate of macrophages on Huh7‐shNC, Huh7‐HBx, Huh7‐HBx‐shNC and Huh7‐HBx‐shGPC3; (E) Live cell imager to observe the process of macrophages phagocytosing Huh7‐shNC, Huh7‐HBx, Huh7‐HBx‐shNC and Huh7‐HBx‐shGPC3.

To determine the key role of N‐GPC3 in HBx‐mediated immune evasion, this study knocked down GPC3 in HBx‐overexpressing Huh7 cells and established four groups for comparison: Huh7‐shNC (control), Huh7‐HBx (HBx overexpression), Huh7‐HBx‐shNC (HBx overexpression + empty vector) and Huh7‐HBx‐shGPC3 (HBx overexpression + GPC3 knockdown). Western blot and flow cytometry confirmed that both N‐GPC3 and total cell surface GPC3 expression were significantly decreased following GPC3 knockdown (Figure 7A,B). After co‐culturing cells from each group with M1‐type macrophages, flow cytometry revealed that the proportion of phagocytosed tumour cells was significantly higher in the Huh7‐HBx‐shGPC3 group compared to the Huh7‐HBx group (Figure 7C). Immunofluorescence results further validated at the morphological level that GPC3 knockdown reversed the inhibition of macrophage phagocytosis caused by HBx (Figure 7D). Long‐term dynamic monitoring using a live‐cell imaging system also demonstrated that macrophage phagocytic activity against the Huh7‐HBx‐shGPC3 group was consistently higher than against the Huh7‐HBx and Huh7‐HBx‐shNC groups (Figure 7E). These results establish N‐GPC3 as a critical downstream effector required for HBx‐mediated immune evasion from macrophage phagocytosis in HCC cells. Knockdown of GPC3 significantly reverses the HBx‐mediated immune evasion phenotype, thereby functionally establishing the necessity of GPC3 in this pathway.

## 4 Discussion

Immune evasion is a core hallmark of malignant tumours. Cancer cells can transmit inhibitory signals to immune cells via immune checkpoints, thereby escaping immune surveillance and even reshaping an immune microenvironment conducive to tumour growth [31, 32] TAMs are the most abundant immune cells in the tumour microenvironment, and their phenotype is closely linked to their function [33] It is widely accepted that M1‐type TAMs typically exert pro‐inflammatory and antitumor functions, whereas M2‐type TAMs promote immunosuppression and tumour progression by secreting anti‐inflammatory factors [34, 35] TAMs are considered one of the most critical nodes for intervention within the immune network, and targeting TAM‐mediated immunosuppression has emerged as a significant therapeutic strategy [36, 37, 38] In this context, our study utilized a co‐culture model of tumour cells and macrophages to investigate the effect of HBx and GPC3 on the phagocytosis by TAMs, in order to better identify novel targets for immune evasion.

HBV has been demonstrated to actively suppress host immune responses, and its encoded HBx protein is closely associated with the state of immune tolerance [39] GPC3, as a glycosylphosphatidylinositol‐anchored proteoglycan on the cell membrane, exists in multiple functionally distinct forms on the cell surface [40] Immunohistochemical analysis of tissue microarrays from 30 HCC patients in this study revealed a significant positive correlation between HBx, GPC3 and the macrophage marker CD86, suggesting a potential association between HBx expression status and both GPC3 levels and tumour‐associated macrophage infiltration. Building on these clinical observations, in vitro experiments demonstrated that HBx selectively enhances the generation and membrane enrichment of N‐GPC3 without altering total GPC3 abundance, indicating regulation at the post‐translational level at p‐ERK levels are significantly correlated with Furin expression [29, 30] we conducted intervention experiments using the p‐ERK inhibitor PD98059 and the agonist CCT020312. The results demonstrated that the p‐EK inhibitor PD98059 blocked the HBx‐mediated upregulation of Furin and N‐GPC3, while the agonist CCT020312 partially restored the levels of p‐ERK, Furin, and N‐GPC3 that were reduced by HBx knockdown. Collectively, HBx enhances GPC3 cleavage through activation of the p‐ERK–Furin axis, thereby governing the generation and functional presentation of membrane‐associated N‐GPC3. These findings elucidate the molecular mechanism by which HBx's regulatory role in immune tolerance is closely linked to its modification of GPC3 isoform expression and membrane presentation.

Tumour immune evasion is a complex process involving interactions among multiple cell types. Macrophages, as key effector cells of innate immunity, play a direct role in tumour clearance through their capacities for recognition, migration and phagocytosis. To investigate the role of HBx in this process, this study established an in vitro co‐culture system of HCC cells and M1‐type macrophages, and employed real‐time live‐cell imaging technology to dynamically monitor intercellular interactions [17] The results demonstrated an inverse association between HBx expression and macrophage phagocytic activity. In co‐cultures with Huh7 cells stably overexpressing HBx, both the phagocytic rate and the mean phagocytic intensity of macrophages against cancer cells were consistently lower than those in the control group. Conversely, in co‐cultures with PLC cells where HBx was knocked down, macrophage phagocytic activity was markedly restored. These experimental findings directly demonstrate that HBx can impair the efficiency of macrophage‐mediated clearance of HCC cells by influencing multiple sequential steps, including cell migration, targeted recognition, and ultimately phagocytosis. As central effectors of innate immune surveillance, macrophages represent a critical barrier to tumour progression. Our findings identify a mechanism by which HBx promotes immune evasion in HCC by suppressing macrophage phagocytic function. However, the specific molecular pathways through which HBx mediates immune evasion have not yet been fully elucidated.

Tumour cells can evade phagocytic clearance by macrophages through the expression of “don't eat me” signalling molecules such as CD47 and PD‐L1. Blockade strategies targeting these signals have demonstrated potential for clinical translation [41, 42, 43, 44, 45] In hepatocellular carcinoma, GPC3 has been reported to recruit M2‐type macrophages and promote tumour progression; however, whether it can function as a “don't eat me” signal to directly regulate macrophage phagocytic activity remains unexplored [22] In this study, we initially discovered that HBx promotes the expression of membrane‐bound N‐GPC3 and significantly inhibits the phagocytosis of HCC cells by macrophages. To further verify whether N‐GPC3 mediates this process, we knocked down GPC3 in HBx‐overexpressing cells and observed that the phagocytosis‐resistant phenotype was significantly reversed. These data indicate that GPC3 is a key downstream effector molecule through which HBx suppresses macrophage phagocytic function. Accordingly, this study reveals for the first time that HBx endows tumour cells with the ability to resist macrophage phagocytosis by upregulating the expression of N‐GPC3 on the HCC cell membrane. This finding not only expands the understanding of HBx's oncogenic mechanism from the perspective of immune evasion but also provides a novel theoretical basis for immunotherapeutic strategies targeting the cleavage process of the GPC3 protein.

This study delineates a central mechanism by which HBx promotes immune evasion in HCC, whereby activation of the MAPK/ERK pathway upregulates Furin to enhance GPC3 cleavage and membrane‐associated N‐GPC3 generation, ultimately suppressing macrophage phagocytosis. However, this study has certain limitations. The experiments were primarily conducted in cell lines with modulated HBx expression; future validation in models with differential GPC3 expression, complemented by in vivo mouse experiments, is necessary to confirm the clinical relevance of this pathway. Nevertheless, despite these areas for improvement, this study provides important insights for HCC immunotherapy. As a liver cancer‐specific marker, the expression level and conformational alteration of GPC3 may remodel immune signals on the tumour cell surface. These findings highlight GPC3 conformational regulation as a promising therapeutic avenue for enhancing immune‐mediated tumour clearance and advancing precision immunotherapy in HCC.

## Author Contributions

**Wei Li:** formal analysis, investigation. **Yuli Zhou:** formal analysis, investigation, resources. **Kaiqi Zhang:** conceptualization, formal analysis, methodology. **Zhongzheng Liu:** conceptualization, resources. **Yaoquan Lin:** formal analysis, investigation. **Hongjiu Yu:** investigation, resources.

## Funding

This work was supported by the Senior Talent Program of Hainan Natural Science Foundation (821RC707), the Postgraduate Innovation Project of Hainan Medical College (Qhys2023‐498).

## Ethics Statement

This study's subjects provided informed consent, and the collection and use of tissue specimens were approved by the Medical Ethics Committee of Hainan Medical University. The Institutional Ethics Committee of Hainan Medical University and the institution review board number was No. HYLL‐2024‐827.

## Consent

All authors have read and agreed to publish this manuscript.

## Conflicts of Interest

The authors declare no conflicts of interest.

## Data Availability

The data that support the findings of this study are available from the corresponding author upon reasonable request.
